# Tolerance of the venom immunotherapy switch from a highly purified aluminium hydroxide adsorbed hymenoptera venom extract to a nonpurified aqueous venom extract

**DOI:** 10.1016/j.waojou.2025.101160

**Published:** 2025-12-22

**Authors:** Anna Gschwend, Lukas Joerg

**Affiliations:** Division of Allergology and Clinical Immunology, Department of Pulmonary Medicine, Allergology and Clinical Immunology, Inselspital, Bern University Hospital, University of Bern, Bern, Switzerland

**Keywords:** Hymenoptera, Venoms, Immunotherapy, Aluminum hydroxide, Drug switching

## Abstract

**Background:**

European guidelines caution against switching from purified aluminium-hydroxide–adsorbed (AHA) venom extracts to non-purified aqueous extracts, and supporting data are lacking.

**Objective:**

To assess feasibility and short-term safety of switching from Alutard® (AHA) to Venomil® (non-purified aqueous) in routine care.

**Methods:**

Retrospective single-centre case series (March 2021–August 2024). Fifteen patients with hymenoptera venom allergy (13 bee, 2 wasp) on maintenance Alutard® were switched to Venomil®. Starting doses and escalation were individualised. Primary outcome was tolerability during switch; secondary outcomes were visits required to re-establish maintenance dosing.

**Results:**

Before switching, 10/15 were on 100 μg and 5/15 on 200 μg. Starting Venomil® doses were 20 μg (7/15), 30 μg (1/15), or 50 μg (6/15); 1 patient underwent ultra-rush re-induction after a systemic field sting on 100,000 SQU Alutard®. Fourteen of 15 patients tolerated the switch without systemic events; 1 bee-venom patient developed a Mueller grade II reaction during escalation toward 100 μg but subsequently reached 200 μg with gradual outpatient increments. Among those without events, 9/13 reached 100 μg on day 1 and 4/13 by day 7; achieving 200 μg typically required 3–4 visits.

**Conclusions:**

In a real-world setting with product constraints, switching from purified AHA to a non-purified aqueous extract was feasible and generally well tolerated when dosing was individualised and monitored. Prospective, standardised studies with sting-challenge endpoints are warranted.

## Background

Non-purified aqueous, purified aqueous, and purified aluminium hydroxide-adsorbed (AHA; “depot”) extracts are used for subcutaneous venom immunotherapy (VIT) in hymenoptera venom allergy (HVA). Availability varies across Europe; in several countries including Switzerland, Alutard® (ALK-Abelló, Hørsholm, Denmark), a purified AHA extract, is the only approved product. Current European guidance notes that aqueous preparations may be preferable in lifelong therapy or when high maintenance doses (eg, 200 μg) are required, largely due to cumulative aluminium exposure considerations in depot products.^1^ High-dose and lifelong AHA-VIT have not been specifically evaluated by the European Medicines Agency (EMA). A 200 μg maintenance dose and/or lifelong therapy is considered in patients with inadequate protection at 100 μg, in high-exposure populations (eg, beekeepers), and in those with risk factors for treatment failure (very severe initial sting reactions, mastocytosis, elevated basal tryptase, or systemic VIT-related reactions).^1^^,^^2^

In Switzerland, treatment with aqueous extracts is feasible only via import and case-by-case insurer approval, a situation mirrored in some other European settings. Supply interruptions have led to temporary switches from aqueous products (Pharmalgen® [discontinued], Venomil® [Bencard], Venomenhal® [HAL]) to Alutard®. Prior studies suggest that switching to purified AHA is generally well tolerated, and switching between aqueous products is feasible—often with dose reductions.^3^, ^4^, ^5^ However, current guidelines provide no supporting evidence for switching from highly purified AHA to non-purified aqueous extracts and caution that non-purified aqueous preparations carry higher rates of local and some systemic reactions, particularly during build-up.

## Methods

We retrospectively reviewed all patients switched from Alutard® to Venomil® between March 2021 and August 2024 in a single allergy center (quality-assurance project). All patients provided informed consent for analysis and publication of their allergy history.

## Cohort

Fifteen patients with HVA were included (13 bee venom [BV], 2 wasp venom). The characteristics of the patients and reasons for the switch are detailed in Table 1. Four patients had never received aqueous extracts before; 1 had prior exposure of varying duration (in 3/11, an aqueous extract was used only on day 1 of ultra-rush induction before conversion to depot on day 7). Before the re-switch, all 11 had received purified AHA for ≥6 months; 3 for ≥12 months and 4 for ≥24 months. The maintenance dose immediately prior to switching was 100 μg in 10/15 (67%) and 200 μg in 5/15 (33%).

**Table 1:** Patient characteristics.

	Bee venom allergy	Wasp venom allergy
	N = 13	N = 2
**Demographics**		
Age	44.07 (±17.8)	65 (±16.9)
Gender (female)	9	1
Gender (male)	4	1
**Grade of index reaction (H.L. Muelle**r):		
Grade I	0	0
Grade II	4	0
Grade III	3	0
Grade IV	6	2
**Cause of change**		
Bee keeper/Increased exposure	10	
Sting not tolerated under Alutard	2	
Mastocytosis/high tryptase	1	2
**Tolerance of subsequent updosing**	12	2
**Total number of change related systemic reactions:**		
Grade of reaction (H.L. Mueller)		
Grade I		
Grade II	1	
Grade III		
Grade IV		

Values are mean ± standard deviation for continuous variables, or median

## Switch protocols

Protocols were individualised as shown in Table 2. In 7/15 (46%) the dose was reduced to 20 μg and then increased in 30-min intervals. One patient (7%) started at 30 μg. Six patients (40%) started at 50 μg. One BV-allergic patient who had sustained a systemic sting reaction while on 100,000 SQU Alutard® underwent full re-induction with Venomil® using an ultra-rush protocol.

**Table 2:** Course of treatment and different immunotherapy protocols for Switch from Alutard® to Venomil

Patient (Gender/Alter)	Venom/Grade (H.L. Mueller)	Tryptase (mcg/L)	Duration the therapy before the change (Months)	Duration of therapy with Depo preparation before the change (Months)	Maintenance Dose before change (SQU)	Maintenance Dose reached at (days)	Protocol (dose in mcg at 30-min intervals)
M18	Bee/III	2.8	72	48	100.000	1	Day 1: 20 + 30+50
F59	Bee/III	3.8	45	24	100.000	1	Day 1: 20 + 30+50
F19	Bee/II	14.2	8	8	100.000	1	Day 1: 20 + 30+50
F52	Bee/II	4.2	19	12	100.000	1	Day 1: 20 + 30+50
F59	Bee/III	4.9	3	3	200.000	21	Day 1: 20 + 30+30 Day 7: 50 + 50 Day 1: 100 + 50 Day 21 : 200
M63	Bee/IV	3.5	40	14	200.000	42	Day 1: 20 + 30 Day 14: 50 + 50 Day 28: 100 + 50 Day 42: 200
M21	Bee/IV	3.6	8	8	100.000	7	Day 1: 20 + 30 Day 7: 50 + 50
M77	Wasp/IV	17.6	60	60	100.000	7	Day 1: 30 + 30 Day 7: 100
F69	Bee/IV	2.3	11	11	100.000	1	Day 1: 50 + 50
F53	Wasp/IV	18.1	64	47	100.000	1	Day 1: 50 + 50
F42	Bee/IV	8	108	6	200.000	1	Day 1: 50 + 50+100
F45	Bee/II	5.8	3	3	100.000	1	Day 1 50 + 50
M46	Bee/IV	2.2	19	16	200.000	14	Day 1: 50 + 25+25 Day 7: 50 + 50+50 Day 14: 100 + 100
F32	Bee/II	<9	9	8	200.000	14	Day 1: 50 + 50 Day 7: 100 + 50 Day 14 : 100 + 100
F48	Bee/IV	5.9	6	6	100.000	42	Day 1: (Ultra-rush) cumulative 100 Day 7: 50 + 50 Day 14: 100 Day 28: 100 + 50 Day 42: 150 + 50

## Tolerability and early outcomes

Fourteen of 15 patients tolerated the switch without systemic events. One BV patient developed a Mueller grade II reaction during escalation from 50 μg toward 100 μg; this patient subsequently achieved the target 200 μg Venomil® dose with gradual weekly increments in the outpatient setting. Notably, the indication for switching in this case was inadequate protection under 100,000 SQU Alutard® (systemic field sting reaction), consistent with guideline-based reasons to consider higher dosing and/or re-induction.

Among those who tolerated the switch uneventfully, 9/13 (69–70%) reached a cumulative 100 μg on day 1; the remaining 4/13 (30–31%) reached 100 μg by day 7. Patients targeting 200 μg generally required 3–4 outpatient visits.

## Limitations

The main limitation of this study is the small sample size and the variability of the switch protocols applied. From a methodological perspective, a more homogeneous approach would have been preferable; however, this would only have been feasible in a prospective design rather than in a retrospective case series. In addition, some patients had previously been treated with aqueous preparations in their past.

## Conclusions

Despite these limitations, this is the first case series to demonstrate the feasibility of switching from highly purified, aluminum-adsorbed venom preparations to unpurified aqueous extracts. The presented protocols may be considered individually in clinical practice when a switch is required, for example in cases of inadequate protection at the standard dose of 100 μg, in high-risk populations, or in patients with additional risk factors for treatment failure. Our data indicate that the switch from Alutard® to an unpurified aqueous venom extract is generally well tolerated, even in high-risk patients (eg, those with mastocytosis, bee venom allergy, or severe systemic sting reactions).^6^

In most cases, the switch can be completed within 1 to 2 outpatient visits. A dose reduction to 50 μg proved safe in the majority of patients, while in high-risk individuals a further reduction to 20 μg should be considered, particularly when the switch is performed due to systemic reactions under Alutard®. In cases of very severe systemic reactions despite adequate treatment with the standard maintenance dose (100 μg = 100,000 SQU Alutard®), re-initiation of updosing with an aqueous extract (eg, via an ultra-rush protocol) may represent a safe and effective alternative.

## Consent for publication

All authors consented for publication.

## Author contribution

AG and LJ designed and planned the project, acquired, analyzed and interpreted the data and wrote the manuscript. AG and LJ participated in data analysis, interpretation, writing the manuscript and critically reviewed. All authors read and approved the final manuscript.

## Ethics approval and consent to participate

All data collection and analysis were conducted as part of a quality assurance project. Additionally, data analysis was approved by the local ethics committee (Kantonale Ethikkommission Bern, Project-ID 2023-01049), and all patients included in this study gave informed consent.

## Availability of materials

Data and materials are available from the corresponding author on reasonable request.

## Declaration of generative AI and AI-assisted technologies in the manuscript preparation process

During the preparation of this work the authors used [ChatGPT, OpenAI] in order to assist with (i) format and word-count optimization, (ii) language and style corrections, and (iii) formatting of references according to the journal's guidelines. The AI was not used to generate substantive content, analyze data, or perform scientific evaluation. All AI-suggested edits were critically reviewed, revised as needed, and approved by the authors; responsibility for the content and its accuracy rests entirely with the authors. No sensitive or identifiable patient data were entered into any AI services.

## Funding

This study was institutional funded.

## Conflicts of interest

The Institution received sponsoring for educational activities from Bencard and ALK. AG received support for attending scientific meeting and travel from ALK, AG received advisory board fees from ALK (in 2020).

## References

[1] Sturm G.J., Varga E.M., Roberts G. (2018). EAACI guidelines on allergen immunotherapy: hymenoptera venom allergy. Allergy.

[2] Sturm G.J., Arzt-Gradwohl L. (2024). An algorithm for the diagnosis and treatment of Hymenoptera venom allergy, 2024 update. Allergy.

[3] Stoevesandt J., Trautmann A. (2019). Lessons from times of shortage: interchangeability of venom preparations and dosing protocols. Allergy.

[4] Scarpone R., Oestmann E., Kraft M., Worm M. (2020). Good tolerability when switching from an aqueous ultra-rush Hymenoptera venom immunotherapy to a depot preparation. Allergy.

[5] Jörg L., Gschwend A., Fricker M., Helbling A. (2021). Tolerance of an immunotherapy switch between two aqueous hymenoptera venoms. Allergy.

[6] Fehr D., Micaletto S., Moehr T., Grendelmeier P.S. (2019). Risk factors for severe systemic sting reactions in wasp (Vespula spp.) and honeybee (Apis mellifera) venom-allergic patients. Clin Transl Allergy.

